# Milena Auretta Rosso first female Italian neurosurgeon

**DOI:** 10.3389/fmed.2026.1712773

**Published:** 2026-02-27

**Authors:** Keyvan Mostofi, Gianluca Caragliano

**Affiliations:** Centre Clinical de Soyaux, Soyaux, France

**Keywords:** European neurosurgery, female neurosurgeons, history of neurosurgery, Milena Auretta Rosso, neurosurgery in Italy

## Abstract

Although the representation of women in the field of surgery is on the rise, significant challenges remain in achieving gender equality, particularly in neurosurgery, a domain that has historically been dominated by men until relatively recently. The advancements made in this area owe much to trailblazing women like Milena Auretta Rosso, who holds the distinction of being Italy’s first female neurosurgeon. It is crucial to acknowledge their contributions, careers, and the adversities they faced in pursuit of their ambitions. Born in 1943 in Genoa, Italy, Rosso graduated with honors in General Medicine from Sapienza University in Rome in 1969. After completing her medical education at the same institution in 1972, she became the first female neurosurgeon in Italy. Throughout her academic journey, she encountered numerous instances of hostility and misogyny. In 1974, an accident resulted in a broken finger on her right hand, which ultimately ended her neurosurgical career. This pivotal moment led her to redirect her professional path while remaining in the medical field, as she pursued studies in iridology. Currently, she practices in Rome, where she also engages in various conferences and has authored a book.

## Introduction

Despite a traditionally patriarchal society, during the Renaissance in Italy, several feminist authors advocated for the concept of gender equality and the necessity of education for women. This notion was particularly significant as Italy experienced the establishment of numerous universities during this period, which did not admit women (1–3). Modesta Pozzo is among these authors, renowned for her prominent work, Il Merito delle donne (The Merit of Women), released in 1600. In this text, she critiques the conduct of men towards women while extolling the virtues and intellect of women, all without asserting the notion of equality between the genders (4). At the onset of the 19th century, a significant portion of peasant women were illiterate (5). The Casati law, enacted in 1859, established a framework for training young women to become teachers in public schools. Furthermore, in 1876, women were permitted to enroll in Italian universities, and the following year, they gained the right to provide testimony in legal matters (6, 7). However, with the rise of Fascism, a deliberate effort was made to reinforce the subservience of women to men, compelling them to prioritize familial and domestic responsibilities (8). Mussolini asserted, “Women must obey. My perspective on her role within the State stands in stark contrast to all forms of feminism. While she should not be treated as a slave, granting her the right to vote would invite ridicule.” In our society, her significance should be minimized (9). Italy’s female employment rate is among the lowest in Europe, standing at 51% for the 20–64 age group, in contrast to the European average of 65%. Furthermore, even when considering equal pay, women continue to earn less than their male counterparts on average (10–12). This disparity is similarly observed within the medical profession concerning female physicians (13). The gender gap continues to persist in the healthcare sector, deeply entrenched in biases and sexist conventions that have existed for millennia (14). A recent survey conducted among Italian female neurosurgeons revealed that a majority, specifically 45%, reported rarely encountering harassment. However, 66% expressed the belief that they were treated differently due to their gender (15, 16). It is noteworthy that Italy has seen female neurosurgeons only since the 1970s, with Milena Auretta Rosso being the first to embark on this career path in 1972 (17). This paper emphasizes the contributions and professional journey of this trailblazer in neurosurgery within her nation. Today, Dr. Rosso expresses deep satisfaction at witnessing the growing presence of women in neurosurgery both in Italy and internationally. She believes that this progress is partly the result of the determination of the pioneering generation of women who challenged entrenched patriarchal norms in surgical training. According to her, the increasing number of female residents reflects the gradual erosion of discriminatory attitudes that once severely limited women’s advancement within the field.

## Life and career

Milena Auretta Rosso was born in Genoa, Italy, on December 30, 1943, during a particularly challenging time in the final years of the Second World War for the residents of Genoa. The city experienced considerable destruction in the final two years of the conflict, emerging from the war with extensive damage, including thousands of civilian deaths and more than 16,000 buildings either destroyed or compromised. Her mother was a homemaker, while her father was employed by the multinational company “Pertusola Sud,” an Italian-British firm based in London with operational facilities in Crotone, specializing in metallurgy and the global export of minerals and metals. Milena has a younger sister employed as an architect in Rome. During her conversation with the authors, Milena reflects on her childhood dream of becoming a neurosurgeon, mentioning that observing surgical procedures in her teenage years strengthened her desire to follow a career in surgery. She attended the Liceo Classico Statale ‘Andrea D’Oria’ in Genoa. In 1962, she commenced her studies at the University of La Sapienza in Rome. At that time, there was no entrance examination, allowing her to enroll directly. The final years of her academic journey coincided with the significant events of 1968. Specifically, from 1967 to 1969, Italy underwent what is referred to as the ‘Italian May Creep.’ Initially, these were student demonstrations opposing a proposed bill that aimed to impose more stringent conditions for university admission. Subsequently, the protests expanded to encompass the entire Italian higher education system, with some demonstrations addressing issues related to gender inequality. Milena describes this era in the following manner: « The limited number of women in the department during that time adhered to a very conservative style, predominantly wearing black attire, with their hair pulled back and devoid of any makeup. In stark contrast, I embodied the essence of an Italian Marilyn Monroe: my platinum blonde hair flowed freely, I wore bold makeup, and I opted for a miniskirt. This was my expression of femininity, which in no way conflicted with the utmost dedication I devoted to my professional responsibilities. It was 1968, a pivotal moment when women were actively shaping a new world, advocating for feminism and women’s rights. » Her academic journey concluded successfully, culminating in her graduation with honors in the Doctor of Medicine program in 1969. She achieved outstanding results in her final examination, scoring 110 out of 110, and was awarded the degree of Doctor of Medicine. She subsequently enrolled in the neurosurgery specialty at La Sapienza University. In 1969, she commenced her internship in neurosurgery at the Medico Interno Neurochirurgo within the Institute of Neurosurgery. As the sole female in her cohort, she became a target for misogynistic and sexist behavior. Her peers would often assert that her place did not belong in the field of neurosurgery. Notably, she was the only female intern in the program. She vividly recalls an incident involving a conspiracy by some interns that nearly jeopardized her life. She recounts the experience as follows: “The incident occurred in the secretariat of the University of Rome ‘La Sapienza,’ where several young specialists were present. They forcibly pushed me towards the window, intending to kiss me to demonstrate where my ‘place as a woman’ was. To evade their aggressive and sexist actions, I had to escape through the window by climbing along the ledge. It was a perilous situation, but I had no alternative. Fortunately, there was a wide ledge leading to another window that provided my escape. It was quite amusing to see my patients watching me swing from one window to another in my white coat, likely thinking I was insane. » The superiors who worked alongside him did not hesitate to express their views on masculinism and sexism. She particularly recalls an incident during a challenging surgical procedure when her superior remarked that her difficulties were due to her being a woman. She describes the experience as follows: During a lower back surgery that proved to be quite complex, I found myself partnered with a professor. It was the height of summer, and throughout the operation, he made sexist comments, suggesting that my struggles stemmed from my gender. I was already under significant stress from the challenging surgical environment, and on top of that, I had to endure his disparaging remarks for hours. After completing the surgery, I encountered him in the secretary’s office and expressed my gratitude. He looked at me with a puzzled expression, seemingly unable to comprehend my appreciation. I then clarified, “Professor, I genuinely thank you because this summer, I was uncertain about my direction and actions, and you provided me with detailed guidance throughout the procedure. » She completed a one-year internship during the final year of her residency at Henri Mondor Hospital in France, with the intention of specializing in neuro-oncology at that institution. Throughout this time, she encountered instances of misogynistic remarks. She recounts her experience as follows: “During my specialist thesis, I spent a year in France under the guidance of a distinguished neurosurgeon. For my thesis, he assigned me to another neurosurgeon responsible for overseeing my work, along with another intern. After facing several delays and periods of inactivity, I approached him to discuss the lack of progress on my thesis. He informed me that due to my gender, he was unwilling to supervise my thesis and preferred to work solely with the other student. Consequently, I had to take the initiative to study independently in the library, collecting all available resources to enhance my grades. I received no support. Even for typing my thesis, I had to seek last-minute assistance from my father’s secretary, who managed to type it for me overnight (as theses were traditionally typed). Ultimately, I submitted everything on time. » In the final months of her academic journey, she returned to the neurosurgery department at La Sapienza, graduating in 1972 with a distinction of 70/70 cum laude in her final Dr. Rosso began her medical studies in 1962 at the University of La Sapienza in Rome and entered neurosurgical training in 1969. She officially started practicing neurosurgery in 1972. Throughout her early career, she trained and worked in several major neurosurgical centres, including the Policlinico Umberto I in Rome, the Hôpital Henri Mondor in Créteil (France), and later the San Filippo Neri Hospital in Rome, where she continued to practice until her accident in 1974. Her contributions during this period included clinical work in cranial and spinal neurosurgery under the supervision of leading neurosurgeons of the time, marking her as a true pioneer in a field that had no female representation in Italy. During her neurosurgical training and early clinical practice, Dr. Rosso was involved in a broad range of cranial and spinal procedures that reflected the case mix of Italian neurosurgical units in the late 1960s and early 1970s. She worked in the service of Professor Beniamino Guidetti in Rome, where she gained exposure to brain tumor surgery, traumatic brain injuries, and degenerative spinal pathologies. She developed a particular interest in vascular neurosurgery, especially in the management of intracranial aneurysms and subarachnoid hemorrhage, which at that time required highly demanding surgical techniques. Notably, these procedures were performed before the widespread adoption of the operative microscope in Italy, meaning that surgeons relied primarily on loupe magnification and direct visualization, making surgical precision even more challenging. During her training in France at Hôpital Henri Mondor, she met Professor Jean-François Hirsch, further expanding her experience in neuro-oncology and complex cranial approaches. She later continued her professional development in Italy under Professor Chiasserini, consolidating her expertise in both elective and emergency neurosurgical cases.

Upon her return to Italy, she spent two years working in the neurosurgery departments of hospitals in Rome (San Filippo Hospital) and Salerno. Unfortunately, she encountered gender discrimination once more. Prior to her tenure in Salerno, she sought an assistant position in Torino, where the supervising physician explicitly informed her that, despite her excellent grades, he could not hire her solely because she was a woman. In 1974, she experienced a tragic incident that significantly altered the course of her life. While walking a strong Doberman, she was holding the leash with her right hand when the dog suddenly jumped, resulting in a broken finger. This injury hindered her ability to perform surgery effectively, leading to a struggle with depression. From 1975 to 1979, she served as a consulting physician at Aied (Associazione Italiana per l’Educazione Demografica) in Rome.

Following this date, she embarked on an extensive journey that spanned from 1979 to 1993 across South Asia, Brazil, and South America. She received training in Iridology and has since been practicing this discipline in Rome, her place of residence. In 2010, she authored a book titled “Che mi lascino in pace (alle pressioni telepatiche),” which was published by Edition ZONA.

## Discussion

**Figure 1:**
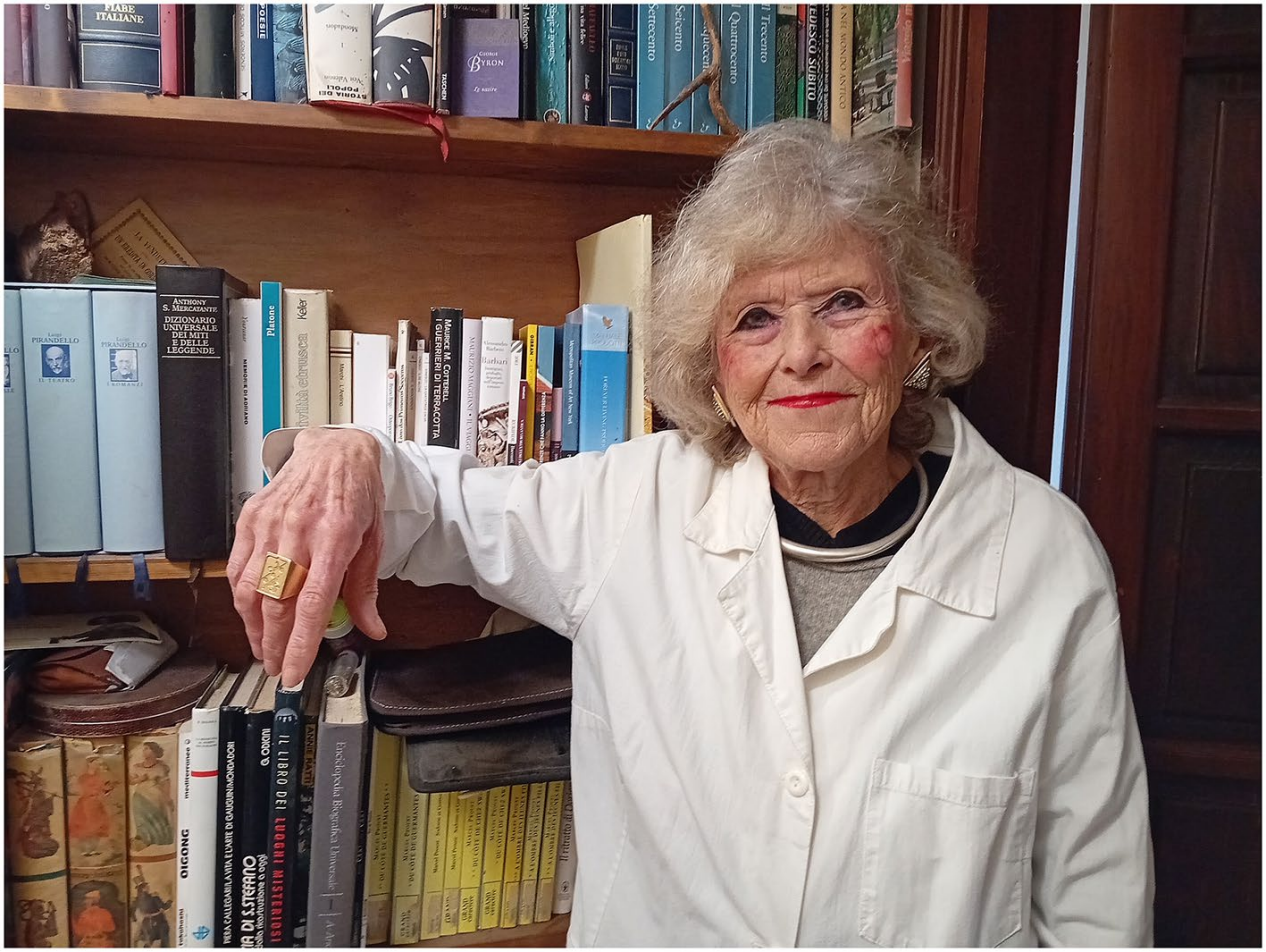
Milena Auretta Rosso.

**Figure 2:**
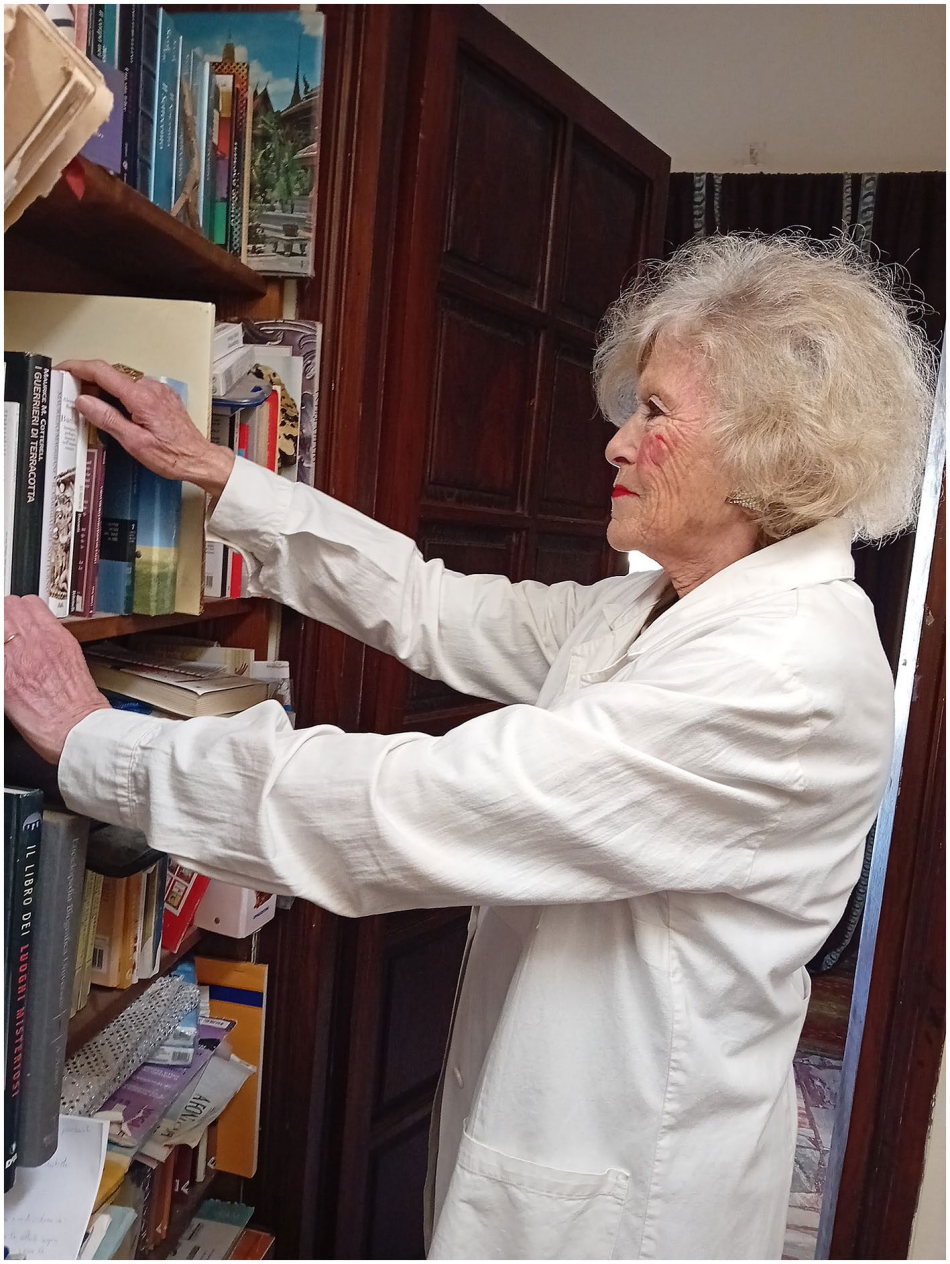
**(a,b)** Milela within her iridology practice.

**Figure 3:**
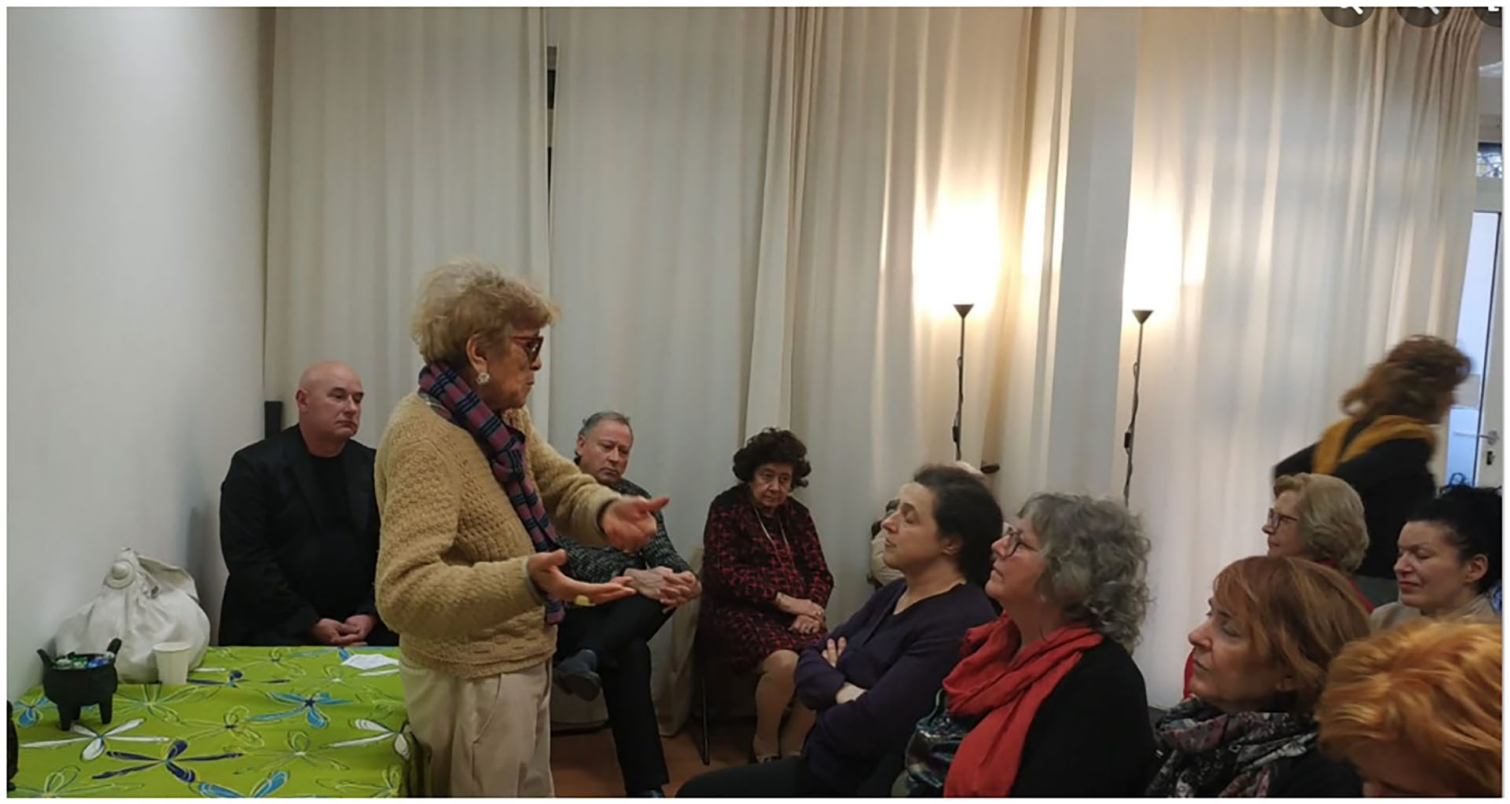
Milena at a conference.

In a seemingly paradoxical way, although women in traditional Italian society played a central role in caring for the sick, assisting childbirth, and preparing medicinal remedies, they were excluded from formal medical practice until the late 19th century. Diagnostic authority and therapeutic responsibility were reserved exclusively for male physicians, who were considered the only legitimate interpreters of scientific knowledge, while women were relegated to subordinate and domestic roles (18). This exclusion was often justified by assumptions of female intellectual inferiority or presumed negligence, which, in turn, legitimized the need for constant male supervision. Although the perception of female healthcare workers evolved after the First World War—particularly with the emergence of nursing as a respected profession—skepticism toward women physicians persisted well into the 20th century (19). Even today, Italy remains among the least feminized medical systems in Western Europe, with only 39.3% of physicians being women (20). A 2019 survey further highlighted that half of Italian doctors had experienced discrimination, while 68% of women reported sacrificing career or family life to meet professional demands (21). Recent trends nevertheless suggest meaningful progress. By 2019, women constituted nearly half of surgical residents (14). Two recent national studies indicate a decline in harassment and overt misogyny among female surgeons and neurosurgeons, who increasingly occupy academic and leadership roles (14, 16). However, these advances must be understood within a historical and cultural context in which women entered neurosurgery only in the 1970s, beginning with Milena Auretta Rosso—Italy’s first female neurosurgeon (22). Neurosurgical training in Italy has undergone significant transformation since the period in which Dr. Rosso completed her specialization. In the late 1960s and early 1970s, neurosurgical education was largely apprenticeship-based, with limited standardization, long working hours, and strong hierarchical structures that often reflected broader societal norms, including gender biases. Technological resources were also limited; for instance, the operative microscope, which later became essential in microneurosurgery, was not yet routinely available in many Italian centers. Over the following decades, training programs became more structured, incorporating national academic regulations, standardized curricula, and increasing international collaboration. The introduction of microsurgical techniques, advanced neuroimaging, and subspecialization has profoundly reshaped both the technical and educational landscape of Italian neurosurgery. These developments have contributed not only to improved patient outcomes but also to a more inclusive professional environment in which women now participate more actively in residency programs, research, and leadership roles (Figures 1–3).

Dr. Rosso’s personal experiences offer unique insight into the cultural barriers that shaped neurosurgical training during this era. Throughout her career, she encountered not only explicit discrimination but also a persistently discouraging environment, marked by comments from male neurosurgeons questioning her competence solely because she was a woman. Such remarks, often delivered during high-stress situations such as complex surgeries, created a hostile workplace that amplified her psychological burden and undermined her sense of belonging. These interpersonal dynamics—ranging from overt misogyny to micro-aggressions—were not isolated events but reflected a broader institutional culture in which women were implicitly discouraged from pursuing neurosurgery. Notably, Dr. Rosso’s reflections today provide valuable historical and contemporary perspective. She expresses deep satisfaction at witnessing the progressive increase in the number of female neurosurgeons in Italy and abroad. According to her, this change reflects a slow but tangible erosion of patriarchal attitudes within surgical training, driven in part by the determination of the pioneering generation of women who challenged exclusionary norms. She perceives current trends—such as the growing presence of women in residency programs and academic neurosurgery—as evidence of cultural evolution, although she believes remaining barriers still require continued institutional awareness. Her case illustrates how structural, cultural, and interpersonal obstacles shaped the trajectory of women in Italian neurosurgery for decades. It also underscores the importance of documenting the experiences of early pioneers whose resilience helped redefine the professional landscape. The historical narrative of Dr. Rosso thus provides not only biographical insight but also a lens through which to interpret the broader dynamics of gender inequality within the surgical professions.

## Conclusion

At a time when the idea of a woman entering neurosurgery was often perceived as incompatible with societal expectations, Milena Auretta Rosso distinguished herself as a groundbreaking figure. Despite confronting overt misogyny, discriminatory barriers, and even episodes that endangered her physical safety, she persisted and demonstrated exceptional professional skill. Although her neurosurgical career was cut short by an accident in 1974, her determination and achievements laid the groundwork for future generations of women in Italian neurosurgery. Her story highlights not only the structural and cultural obstacles that shaped gender inequality in the field, but also the transformative impact of pioneers whose resilience helped reconfigure the landscape of neurosurgical practice in Italy.

## Data Availability

The original contributions presented in the study are included in the article/supplementary material, further inquiries can be directed to the corresponding author.
